# What Do Germans Want to Know About Skin Cancer? A Nationwide Google Search Analysis From 2013 to 2017

**DOI:** 10.2196/10327

**Published:** 2018-05-02

**Authors:** Stefanie Seidl, Barbara Schuster, Melvin Rüth, Tilo Biedermann, Alexander Zink

**Affiliations:** ^1^ Klinikum rechts der Isar Department of Dermatology and Allergy Technical University of Munich Munich Germany

**Keywords:** skin cancer, melanoma, nonmelanoma skin cancer (NMSC), Google, search analysis, population

## Abstract

**Background:**

Experts worldwide agree that skin cancer is a global health issue, but only a few studies have reported on world populations’ interest in skin cancer. Internet search data can reflect the interest of a population in different topics and thereby identify what the population wants to know.

**Objective:**

Our aim was to assess the interest of the German population in nonmelanoma skin cancer and melanoma.

**Methods:**

Google AdWords Keyword Planner was used to identify search terms related to nonmelanoma skin cancer and melanoma in Germany from November 2013 to October 2017. The identified search terms were assessed descriptively using SPSS version 24.0. In addition, the search terms were qualitatively categorized.

**Results:**

A total of 646 skin cancer-related search terms were identified with 19,849,230 Google searches in the period under review. The search terms with the highest search volume were “skin cancer” (n=2,388,500, 12.03%), “white skin cancer” (n=2,056,900, 10.36%), “basalioma” (n=907,000, 4.57%), and “melanoma” (n=717,800, 3.62%). The most searched localizations of nonmelanoma skin cancer were “nose” (n=93,370, 38.99%) and “face” (n=53,270, 22.24%), and the most searched of melanoma were “nails” (n=46,270, 70.61%) and “eye” (n=10,480, 15.99%). The skin cancer‒related category with the highest search volume was “forms of skin cancer” (n=10,162,540, 23.28%) followed by “skin alterations” (n=4,962,020, 11.36%).

**Conclusions:**

Our study provides insight into terms and fields of interest related to skin cancer relevant to the German population. Furthermore, temporal trends and courses are shown. This information could aid in the development and implementation of effective and sustainable awareness campaigns by developing information sources targeted to the population’s broad interest or by implementing new Internet campaigns.

## Introduction

The incidence of skin cancer, including nonmelanoma skin cancer (NMSC) and melanoma, is a major public health issue [1]. In fact, NMSC is the most common cancer among Caucasians worldwide [2]. In Germany alone, approximately 180,000 new cases are reported each year [3]. The main risk factor for NMSC is exposure to solar ultraviolet radiation (UVR) [4]. Thus, sun-exposed skin areas, such as the nose, neck, head, and face, are particularly at risk [5,6]. Less commonly, NMSC can occur on skin areas that are not exposed to sunlight owing to other etiopathogenetic factors, such as alterations in lymphatic circulation [7,8]. Currently, NMSC has an enormous socioeconomic impact with a continuously increasing incidence within the last few years [9]. In Germany, the incidence is estimated to double by 2030 [3]. Not as frequent, but with a substantially higher mortality than NMSC, is melanoma, which is diagnosed in approximately 21,000 new patients each year in Germany [10]. Recently, new diagnostic and treatment options, especially for melanoma, have had a significant impact in the global medical community [11-14]. At the same time, NMSC has been recognized as an occupational disease in some countries, and new techniques and products for UVR protection, including apps and gadgets, have been developed [15,16]. In summary, experts agree about the global problem of skin cancer, but few studies have focused on the interest of the population [17], which might help lower the global burden. One option for analyzing the interest of the population is an Internet search analysis. This is a promising approach to reflecting the population’s interest in a certain topic [18]. It is a novel tool to estimate the impact of disease in a population where traditional methods are inadequate or in the absence of data sources [19]. Analysis of the Internet search volume for different search terms provides insight into the population’s general interest. Therefore, the term “search volume” concentrates on the number of searches of a particular topic or search term. This procedure has been used by communication media for several years [20]. The Internet’s emerging role as a main source of health information for the population [21,22] has created a corresponding value as a novel and informative method in the medical field. Recently, Huang et al reported a small association between online cancer-related information searching behaviors and skin cancer incidences [23], and Wehner et al successfully demonstrated that Internet search volume positively correlated with the incidence and mortality rates of common cancers, including melanoma, in the United States [18]. This indicates that the unconventional method of Internet search analysis can provide useful data on the characteristics of a disease including incidence and mortality. Hence, the Internet search data reflecting the population’s interest can provide information about real-life skin cancer incidence as well as medical needs. For this purpose, the Google search engine is promising for Germany because of its 95% market share [24]. Moreover, the population prefers the use of search engines such as Google over specialized websites when searching for health information online [25-27].

The aim of this study was to analyze the interest of the German population in skin cancer by analyzing Google searches of terms related to NMSC and melanoma.

## Methods

### Study Design

In this retrospective longitudinal study, we used Google AdWords Keyword Planner to identify the search volume of terms related to NMSC and melanoma. Google AdWords Keyword Planner is usually used by advertisers to optimize Google marketing campaigns. The most important part of Google AdWords is keywords. With their help, an advertiser can specify in advance that an ad should be displayed only in the result for a search for the mentioned terms or thematically appropriate pages. Therefore, the tool can search for keyword ideas; new keywords can be included or lists of keywords can be uploaded [28]. However, because this technology indicates the monthly search volume estimated by Google, it can also be used for scientific questions. In our study, the search terms related to NMSC and melanoma were identified using a keyword cluster including the following 13 common terms for skin cancer in German: “skin cancer,” “black skin cancer,” “white skin cancer,” “light skin cancer,” “nonmelanocytic skin cancer,” “nonmelanoma skin cancer,” “NMSC,” “melanoma,” “malignant melanoma,” “basalioma,” “basal cell carcinoma,” “spinalioma,” and “squamous cell carcinoma.” On the basis of this cluster, Google AdWords Keyword Planner identified search terms to be analyzed regarding their search volume. The search volume data included only those from Google users with a German Internet protocol address and were analyzed from November 2013 to October 2017.

**Figure 1:**
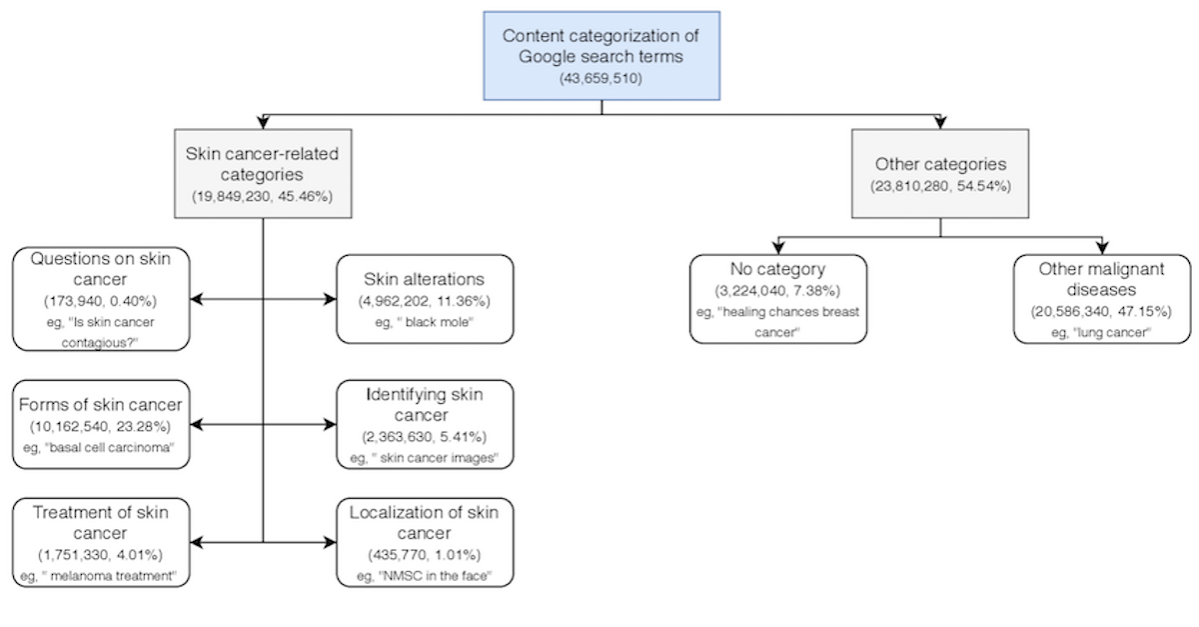
Content categorization of search terms identified by Google AdWords Keyword Planner. The search terms were manually screened and categorized into 6 skin cancer–related categories and 2 other categories. “No category” was used for search terms not fitting in any category. The figure includes absolute numbers and percentages of search volume and an example search term for each category.

### Statistical Analysis

The search volume data from the identified search terms were assessed descriptively using SPSS version 24.0. Furthermore, the search terms were qualitatively categorized based on their content after having been read carefully. In the first step, the content of all search terms was analyzed, and 6 skin cancer-related categories were identified (Figure 1). A further category for “other malignant diseases” (eg, “lung cancer”) was also identified. Search terms that did not fit in any of these categories were placed in the “no category” (eg, “healing chances breast cancer”; Figure 1). Each search term was only assigned to one category. In the second step, we categorized and analyzed the search terms within the category “localization of skin cancer” according to the exact localization of NMSC and melanoma separately.

## Results

In total, Google AdWords Keyword Planner identified 714 search terms related to NMSC and melanoma with a search volume of 43,659,510 in Germany from November 2013 to October 2017. Sixty-eight search terms referred to other malignant diseases or were not assignable terms, while 646 search terms directly referred to skin cancer (Figure 1). Of the skin cancer–related search terms, the most common terms were “skin cancer” (n=2,388,500, 12.03%) followed by “white skin cancer” (n=2,056,900, 10.36%), “basalioma” (n=907,000, 4.57%), “melanoma” (n=717,800, 3.62%), and “black skin cancer” (n=649,400, 3.27%; Table 1 and Multimedia Appendix 1).

### Time Analyses

**Figure 2:**
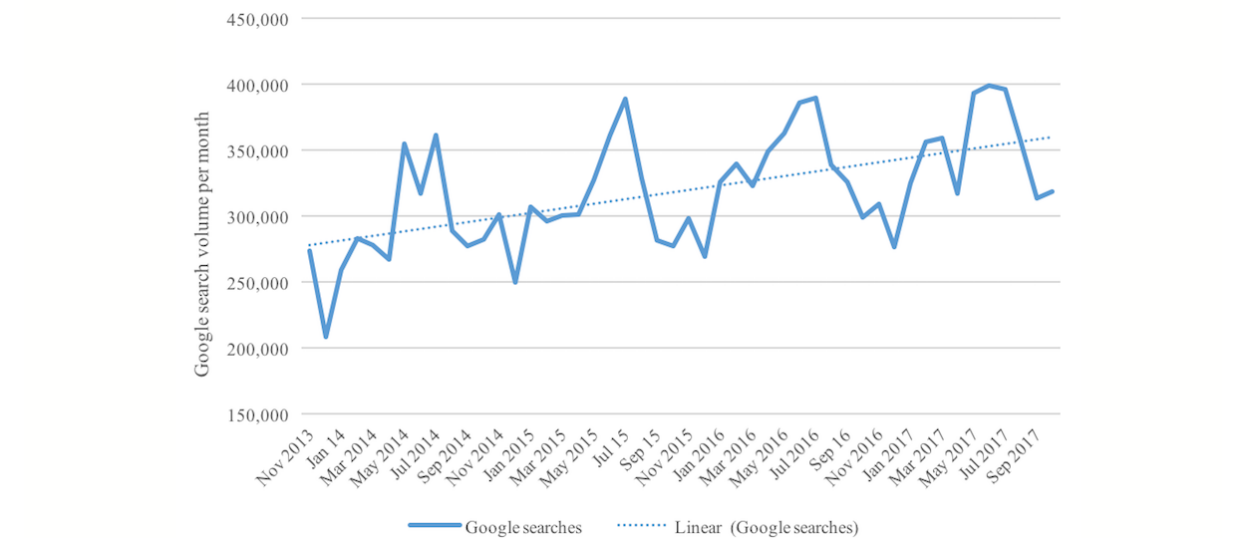
Nationwide Google searches of terms related to nonmelanoma skin cancer and melanoma in Germany from November 2013 to October 2017. Only skin cancer–related search terms are included; search terms from the categories “no category” and “other malignant diseases” are not.

Figure 2 shows the Google search volume of terms related to NMSC and melanoma from November 2013 to October 2017 with annual increases. The lowest search volume was in December 2013 (n=208,400, 1.05%), while the highest volume was in June 2017 (n=398,590, 2.01%). Every year, the search volume increased in April and May, remained high over June and July, and decreased again in August. The month with the highest search volume over all consecutive years was July, except in 2017, when it was June. The largest increase was measured from April 2014 to May 2014, when the search volume increased 32.9% from 266,970 to 354,780 (Figure 2).

**Table 1:** Most frequently searched skin cancer–related terms in Germany from November 2013 to October 2017 (N=19,849,230).

Ranking	Skin cancer–related term	Search volume, n (%)
1	Skin cancer	2,388,500 (12.03)
2	White skin cancer	2,056,900 (10.36)
3	Basalioma	907,000 (4.57)
4	Melanoma	717,800 (3.62)
5	Black skin cancer	649,400 (3.27)
6	Actinic keratosis	482,500 (2.43)
7	Squamous cell carcinoma	433,300 (2.19)
8	Skin cancer pictures	366,700 (1.85)
9	Malignant melanoma	336,900 (1.70)
10	Basal cell carcinoma	292,500 (1.47)

### Nonmelanoma Skin Cancer and Melanoma

**Figure 3:**
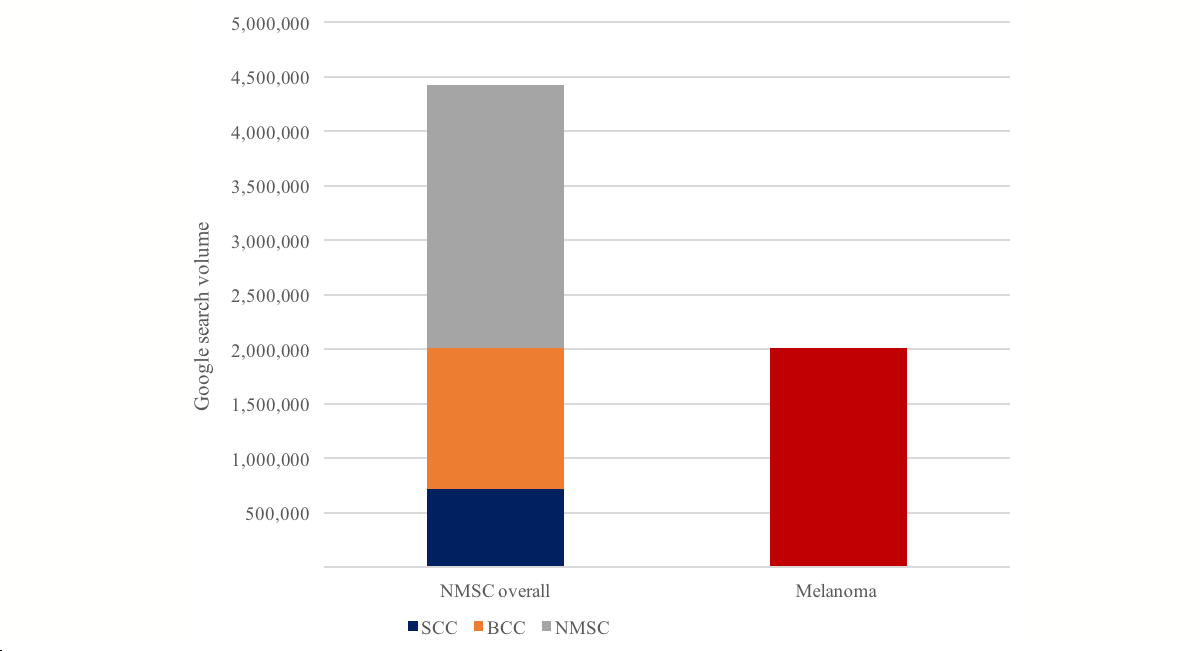
Google search volume of terms related to nonmelanoma skin cancer (NMSC) and melanoma in Germany from November 2013 to October 2017. “NMSC overall” includes search terms such as “white skin cancer”, “BCC”, and “SCC”. “Melanoma” includes search terms such as “malignant melanoma”. (BCC=basal cell carcinoma; SCC=squamous cell carcinoma).

In a total of 8,953,870 searches of terms related to NMSC and melanoma, NMSC-related terms (eg, “white skin cancer,” “nonmelanoma skin cancer”) had a search volume of 4,421,480 (49.39%), which was categorized as referring to basal cell carcinoma or squamous cell carcinoma or not classifiable to either. Melanoma-related terms (eg, “black skin cancer,” “malignant melanoma”) had a search volume of 2,014,130 (22.49%; Figure 3). A total of 2,518,260 (28.12%) searches referred to skin cancer overall, including search terms such as “cancer skin.”

A total of 927,090 searches referred to precursor lesions of skin cancer: 895,710 (96.62%) on NMSC precursor lesions and 7080 (0.76%) on melanoma precursor lesions. A total of 24,300 (2.62%) searches referred to precursor lesions of skin cancer overall (eg, “skin cancer precursor lesion”).

Few searches referred to skin cancer stages (n=204,510) or metastasis of skin cancer (n=58,210); however, melanoma (n=79,830, 39.03% and n=28,170, 48.39%, respectively) had a larger search volume than NMSC (n=63,000, 30.81% and n=15,880, 27.18%, respectively).

### Search Term Categories

In total, 92.62% of the search volume fit into a specific category, whereas 7.38% was summarized into “no category” because of nonspecificity (eg, “cancer”) or the topic (eg, “healing chances breast cancer”).

Nearly half of the search volume (N=43,659,510) was in the category “other malignant diseases” (n=20,586,340, 47.15%) followed by the categories “forms of skin cancer” (n=10,162,540, 23.28%) and “skin alterations” (n=4,962,020, 11.36%; Table 2). Few searches referred to “treatment of skin cancer” (n=1,751,330, 4.01%), “localization of skin cancer” (n=435,770, 1.01%), or “questions on skin cancer” (n=173,940, 0.40%).

### Localization

In a total of 435,770 searches of terms related to skin cancer localization, 239,530 (54.97%) referred to NMSC and 65,530 (15.04%) referred to melanoma. A total of 130,710 searches (29.99%) did not fit into either NMSC or melanoma because the search terms contained only “skin cancer”; these were included in “skin cancer overall.”

**Figure 4:**
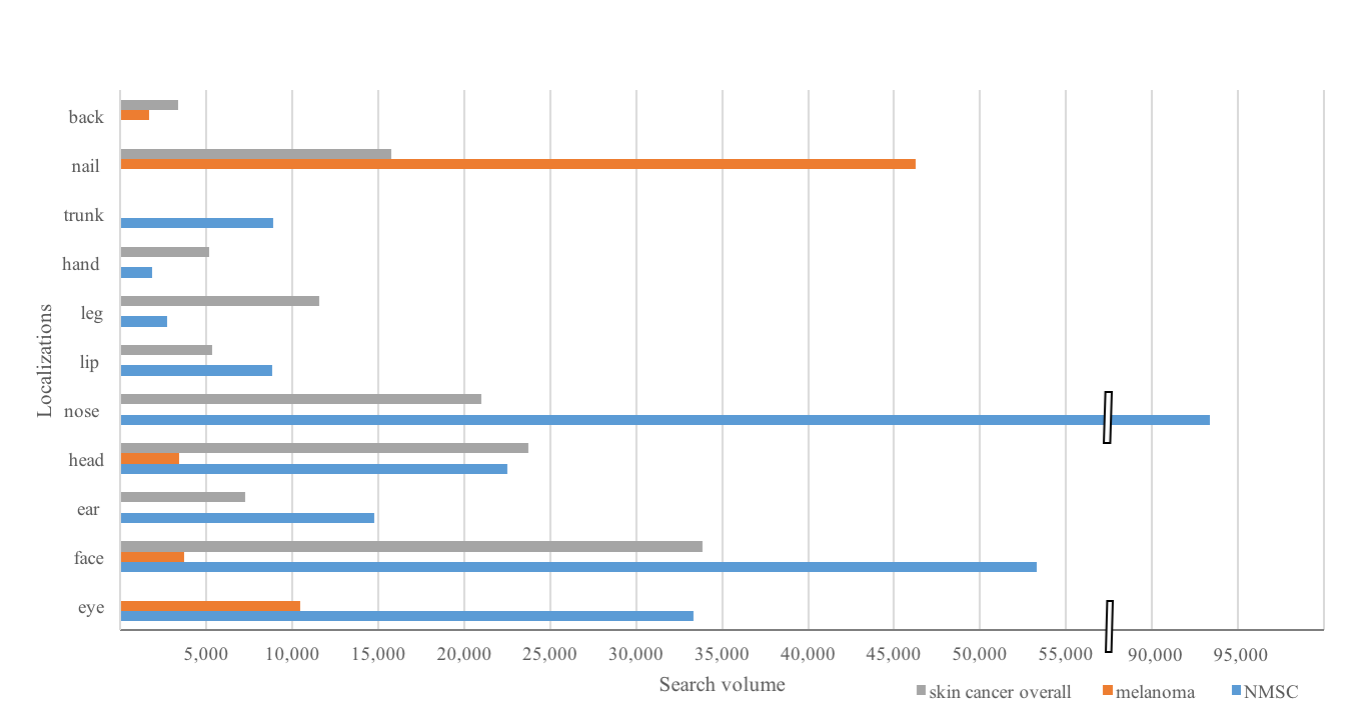
Google search volume of localization of nonmelanoma skin cancer, melanoma, and skin cancer overall in Germany from November 2013 to October 2017. Search terms without an exact skin cancer term (eg, “skin cancer nose”) are included in “skin cancer overall.”.

The most commonly searched localizations of NMSC were “nose” (n=93,370, 38.99%) followed by “face” (n=53,270, 22.24%) and “eye” (n=33,320; 13.9%). There were considerably less NMSC searches for “legs” (n=2730, 1.14 %) or “hands” (n=1860, 0.78%). For melanoma, interestingly, the most commonly searched localization was “nails” (n=46,270, 70.61%). With a search volume of 10,480 (15.99%), “melanoma eye,” which probably refers to uveal melanoma, was the second most searched term, followed by melanoma of the “face” (n=3720, 5.68%) and melanoma of the “head” (n=3420, 5.22%). Few searches were done for melanoma of the “back” (n=1640, 2.50%). For “skin cancer overall,” “face” (n=33,830, 25.88%), “head” (n=23,740, 18.16%), and “nose” (n=20,960, 16.04%) were the most frequently searched localizations (Figure 4).

**Table 2:** Content categorization of the identified search terms related to nonmelanoma skin cancer and melanoma in Germany from November 2013 to October 2017 (N=43,659,510).

Search term category	n (%)
Other malignant diseases	20,586,340 (47.15)
Forms of skin cancer	10,162,540 (23.28)
Skin alterations	4,962,020 (11.36)
No category	3,224,040 (7.38)
Identifying skin cancer	2,363,630 (5.41)
Treatment of skin cancer	1,751,330 (4.01)
Localization of skin cancer	435,770 (1.01)
Questions on skin cancer	173,940 (0.40)

## Discussion

### Principal Considerations

In total, approximately 19.85 million skin cancer-related Google searches were conducted in Germany between November 2013 and October 2017. The search terms with the highest search volume were “skin cancer,” “white skin cancer,” “basalioma,” “melanoma,” and “black skin cancer.” The course of time shows an overall increase in Google search volume from 2013 through 2017, with a higher search volume during each summer.

The overall increase in Google search volume of “NMSC” and “melanoma” in the course of time might be explained, in part, by the continuously increasing incidence of skin cancer [10,29] and public awareness campaigns [30]. This might have also led to an overall increase in public interest in skin cancer. Particularly, the recognition of NMSC as an occupational disease in Germany in 2015 could have led to an increased interest [15,29,31-33], which might be a reason for the higher search volume over summer 2015 compared with summer 2014.

Due to the fact that UVR is the main risk factor for NMSC and a significant risk factor for melanoma [10], it is not surprising that the summer months had the highest search volume each year. In addition, the media report on sun safety and many skin safety campaigns run during this season [34,35]. Moreover, Figure 2 demonstrates that the public interest increases in late spring, when people more frequently wear short-sleeved clothing due to rising temperatures and decreases in late summer when temperatures begin to decrease in Germany [36]. The seasonal variation may also be affected by diagnoses of NMSC and melanoma, which are reported to be significantly higher in late spring and early summer [37,38]. These seasonal differences in search volume were also reported by Bloom et al with regard to skin cancer and melanoma in the United States [17].

The incidence of NMSC is several times higher than that of melanoma [39]. The population’s interest, as measured by the Google search volume, does not reflect this; the search volume of NMSC was not even three times higher than that of melanoma (Figure 3). The small difference might be traced back to the fact that melanoma is associated with a higher mortality, leading to a relatively higher interest. A striking finding is the search volume of precursor lesions of NMSC compared with melanoma. This is probably related to the clearer definition of precursor lesions of NMSC. Moreover, the search volume of metastasis of NMSC is appreciably lower than that of melanoma, which reflects the lower incidence of NMSC metastasis relative to melanoma metastasis [40,41].

The large share of the category “other malignant diseases” can be explained by the fact that it covered several cancers (eg, lung cancer, cervical cancer), while the category “forms of skin cancer” referred only to skin cancer. These other malignant diseases were identified because Google AdWords Keyword Planner also shows search terms that are not primarily relevant to the topic of interest. The search volumes of localizations of NMSC and melanoma reflect the typical localizations where skin cancer is diagnosed [42-44]. The second most searched localization of melanoma, “eye,” gives rise to the question of whether the search was intended for uveal melanoma. Because uveal melanoma is rare [45], our study shows a conspicuously high overall interest, which might reflect people’s special fear or dismay regarding melanoma of the eye.

### Limitations

This study has some limitations. Although Google has a market share of 95% in Germany [24], it is not used by all population groups to the same extent [46]. Younger-age groups use the Internet more frequently than older ones [46], who are more often affected by NMSC [10]. This could have led to underestimation of specific terms that would be searched by affected people. In addition, Google AdWords Keyword Planner gives only estimations regarding the monthly search volume of search terms based on a Google algorithm without any further information. Therefore, we do not know how precise the estimates are. However, it has been shown that Google data do correlate with skin cancer prevalence [18]. Moreover, the automatic completion of search terms, which is suggested by Google, may influence people’s search behavior. It is possible that often-searched terms are even more frequently searched, while less frequently searched terms are discarded. However, this should not have a great impact on our assessment of the overall interest in a particular topic, as interest was mostly assessed in categories rather than by individual search terms.

### Conclusion

This analysis of Google search data reflects the German population’s interest in NMSC and melanoma. Despite the limitations, our study provides insight into terms and fields of interest related to skin cancer relevant to the German population. This information could aid in the development and implementation of effective and sustainable awareness campaigns. Therefore, future sources of information should be developed on the basis of general interest, such as the interest in melanoma of the eye. Educators should also use this technique to identify keywords to use when buying Google ads for Internet campaigns. In addition, prevention and screening programs should take advantage of the increasing role of the Internet as a source for skin cancer‒related information. To facilitate better understanding of Internet search data, the authors’ future studies will focus on the correlation between search volume and real-life characteristics of skin cancer.

## Appendix

Multimedia Appendix 1 The 50 most frequent skin cancer-related search terms in German.

## Abbreviations

NMSC: nonmelanoma skin cancer
UVR: ultraviolet radiation
