# Supplementary material for: What Do Germans Want to Know About Skin Cancer? A Nationwide Google Search Analysis From 2013 to 2017
Source: J Med Internet Res. 2018 May 2;20(5):e10327. doi: 10.2196/10327 (PMC5956155; doi:10.2196/10327)
Supplement: Multimedia Appendix 1 [file jmir_v20i5e10327_app1.pdf]

	Search term
1	Hautkrebs
2	Weißer Hautkrebs
3	Basaliom
4	Melanom
5	Schwarzer Hautkrebs
6	Aktinische Keratose
7	Plattenepithelkarzinom
8	Hautkrebs Bilder
9	Malignes Melanom
10	Basalzellkarzinom
11	Weißer Hautkrebs Bilder
12	Hautkrebs Symptome
13	Hautveränderungen
14	Spinaliom
15	Morbus bowen
16	Hautkrebsscreening
17	Hautkrebs Anzeichen
18	Keratose
19	Hautkrebs erkennen
20	Heller Hautkrebs
21	Hautkrebsvorsorge
22	Basaliom Bilder
23	Schwarzer Hautkrebs Bilder
24	Wie sieht Hautkrebs aus
25	Melanom Bilder
26	Symptome Hautkrebs
27	Hauttumor
28	Aktinische Keratose Bilder
29	Hautkrebsarten
30	Weißer Krebs
31	Hautkrebs weiß
32	Weißer Hautkrebs Symptome
33	Hautkrebs Anfangsstadium
34	Hautkrebs Behandlung
35	Stachelzellkrebs
36	Weißer Hautkrebs Fotos
37	Schwarzes Muttermal
38	Bilder Hautkrebs
39	Spinozelluläres Karzinom
40	Basaliom Nase
41	Weißer Hautkrebs Aussehen
42	Weißer Hautkrebs Anfangsstadium
43	Keratosen
44	Bilder weißer Hautkrebs
45	Weiser Hautkrebs
46	Schwarzer Hautkrebs Lebenserwartung
47	Melanome
48	Weißer Hautkrebs Behandlung
49	Noduläres Melanom
50	Hautkrebs Muttermal
